# Near-Infrared 808 nm Light Boosts Complex IV-Dependent Respiration and Rescues a Parkinson-Related *pink1* Model

**DOI:** 10.1371/journal.pone.0078562

**Published:** 2013-11-11

**Authors:** Melissa Vos, Blaise Lovisa, Ann Geens, Vanessa A. Morais, Georges Wagnières, Hubert van den Bergh, Alec Ginggen, Bart De Strooper, Yanik Tardy, Patrik Verstreken

**Affiliations:** 1 VIB Center for the Biology of Disease, Leuven, Belgium; 2 KU Leuven, Department of Human Genetics and Leuven Research Institute for Neuroscience and Disease (LIND), Leuven, Belgium; 3 Medos International Sàrl, Le Locle, Switzerland; 4 Swiss Federal Institute of Technology (EPFL), Medical Photonics Group, Institute of Chemical Sciences and Engineering (ISIC), Lausanne, Switzerland; 5 Corporate Office of Science & Technology, Johnson & Johnson Services, Inc., New Brunswick, New Jersey, United States of America; Columbia University, United States of America

## Abstract

Mitochondrial electron transport chain (ETC) defects are observed in Parkinson’s disease (PD) patients and in PD fly- and mouse-models; however it remains to be tested if acute improvement of ETC function alleviates PD-relevant defects. We tested the hypothesis that 808 nm infrared light that effectively penetrates tissues rescues *pink1* mutants. We show that irradiating isolated fly or mouse mitochondria with 808 nm light that is absorbed by ETC-Complex IV acutely improves Complex IV-dependent oxygen consumption and ATP production, a feature that is wavelength-specific. Irradiating *Drosophila pink1* mutants using a single dose of 808 nm light results in a rescue of major systemic and mitochondrial defects. Time-course experiments indicate mitochondrial membrane potential defects are rescued prior to mitochondrial morphological defects, also in dopaminergic neurons, suggesting mitochondrial functional defects precede mitochondrial swelling. Thus, our data indicate that improvement of mitochondrial function using infrared light stimulation is a viable strategy to alleviate *pink1*-related defects.

## Introduction

Parkinson’s disease (PD) is a common neurological disease and patients are characterized by progressive dysfunction of the dopaminergic system, explaining motor symptoms [1]. Current therapeutic strategies are mostly focused on managing the symptoms via restoration of dopaminergic tonus using pharmacological agents or via deep brain stimulation [2]. However, the beneficial effects of these strategies are limited in time and significant side-effects have been reported [2]–[4]. Hence, new methodologies that prevent or delay neuronal dysfunction in PD are eagerly awaited.

Environmental factors as well as loss-of-function of several PD-related genes in flies and mice indicate that mitochondrial defects might be a rather common problem in the disease process [5]–[11]. Hence, alleviating mitochondrial defects may be a promising strategy for those PD patients that suffer from mitochondrial deficits. Pink1 is a mitochondrial serine/threonine kinase mutated in PD and loss of Pink1 in different species results in mitochondrial failure [5], [7], [12], [13]. *Pink1* mutant fruit flies show, along with locomotion defects, disrupted mitochondrial membrane potential, reduced ATP levels, reduced Complex I activity and abnormal mitochondrial morphology and swelling [7], [12], [13]. Based on molecular studies and genetic interactions, Pink1 has been suggested to act at different levels; the protein is thought to regulate mitochondrial quality control and autophagy together with Parkin [14]–[18], and it has also been implicated in the control of mitochondrial function by regulating the electron transport chain [5], [7], [11], [19]. However, the relative contribution of these pathways to Pink1–related pathology remains unclear [11], [20]–[22] nor is it known whether acutely improving ETC function is a valuable therapeutic approach for PD patients that suffer from Pink1-induced mitochondrial dysfunction.

The application of long wavelength light (referred to as photobiomodulation or PBM) is thought to exert beneficial effects in wound healing, stroke, optic axonal degeneration and ischemic heart injury [23]–[25]. Furthermore, PBM has also been shown to exert a protective effect on cells [26] and it was shown to protect dopaminergic neurons to MPTP-induced toxicity in rodents [27]. However, the mechanism by which PBM exerts these beneficial effects has remained largely elusive, although an effect on mitochondrial function has been suggested [28]. Given that Parkinson disease fly models display mitochondrial disfunction, we tested the hypothesis that long wavelength 808 nm monochromatic light that is absorbed by Complex IV in cells [28], improves ETC function and can rescue mitochondrial and organismal defects in adult *Drosophila pink1* mutants. While infra-red light effectively penetrates flies, such a strategy may harbor therapeutic potential in patients as well, provided it is combined with an implantable device to locally deliver the light.

## Materials and Methods

All animal experiments were conducted with the approval of the KU Leuven ethics committee.

### Flies and Irradiation

Animals were grown on standard cornmeal and molasses medium. *Pink1* null mutants were *w pink1^B9^* and controls were *w pink1^RV^*. *park* null mutants were *w park^1/Δ21^* and controls were *park^RV^*. *Pink1^B9^*, *pink1^RV^*, *park^1^* and *park^RV^* controls were from Jeehye Park and Jongkyeong Chung (KAIST) (Park et al, 2006), *park^Δ21^* mutant flies were a gift from Graeme Mardon (BCM) (Pesah et al, 2004). *Drp1* mutants were *y w eyFlp GMR::LacZ; p(y^+^) drp1^1/2^ FRT40A* from Hugo Bellen (BCM) and controls were *y w eyFlp GMR::LacZ; p(y^+^) FRT40A.* Mutant animals were selected by the absence of the GFP-marked balancers.

**Figure 1:**
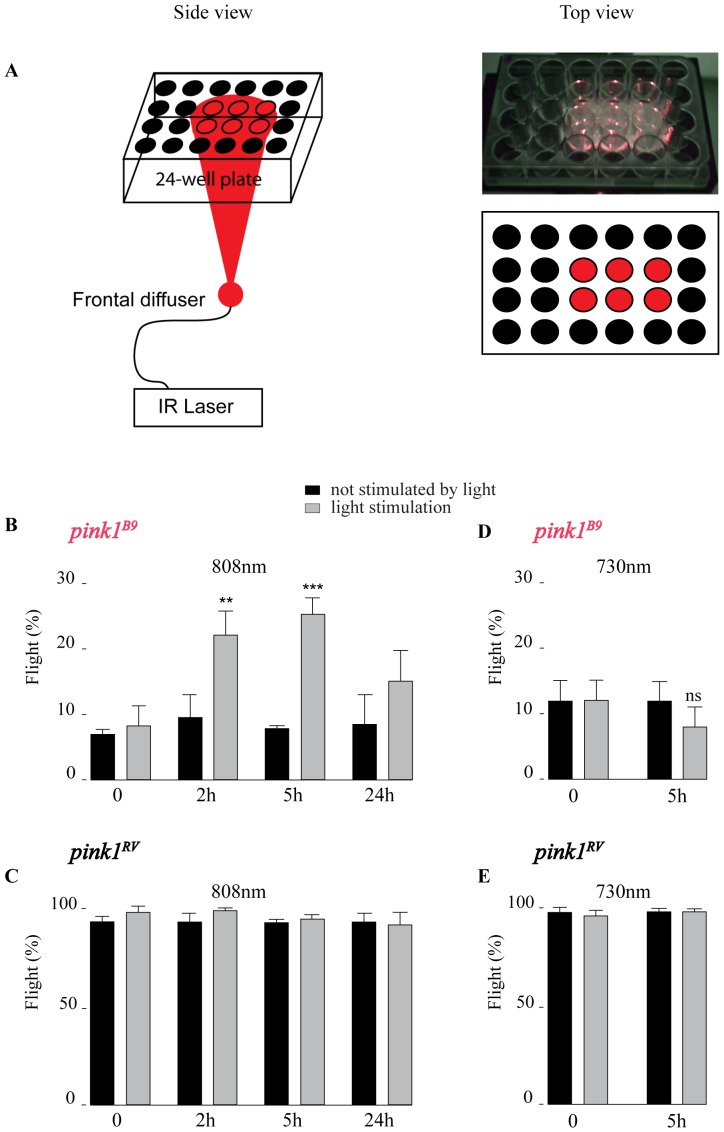
808*pink1^B9^* flight defects. A- Experimental set-up used to illuminate groups of flies using the described laser diodes (see Materials and Methods). ‘red’ wells are illuminated (light stimulation), ‘black’ wells are not (control). B–E Flight ability of 5-day old *pink1^B9^* mutants (B, D) and controls (C, E) illuminated with 808 nm (B–C) or 730 nm (D–E) (grey bars) (100 s at 25 mW/cm^2^) and not-illuminated (black bars) tested at the times indicated after illumination. n = 10 times 5 flies per condition; data represent percentages ± SEM. ANOVA/Dunnet: **: p<0.01; ***: p<0.001; ns = not significant.

For illumination, animals were placed in a 24-well plate (Thermo Scientific BioLite, Langenselbold, Germany), and covered with the plate lid. The plates were bottom-illuminated using continuous lasers that were coupled to a frontal light distributor to homogenize the illumination spot (FD1, Medlight SA, Ecublens, Switzerland). The output power was measured at the distal tip of the light distributor with the help of a powermeter (1918-R, Newport, Darmstadt, Germany), equipped with a thermopile sensor (818P-010-12, Newport, Darmstadt, Germany). Experiments were performed with an 808 nm-GaAs laser diode (RLTMDL-808-2W with a PSU-LED power supply, Roithner Lasertechnik GmbH, Vienna, Austria) or with a 730 nm laser diode (LTL730S with a ADR-1805 driver, Leading-Tech Laser Co. LTD, Shanghai, China). For the 808 nm laser, we tested different illumination irradiances (10–25 mW/cm^2^), illumination durations (25–200 s) and delays between illumination and observation (15 min –24 h) but found that an irradiance of 25 mW/cm^2^ during 100 s, which corresponds to a light dose of 2.5 J/cm^2^, and an incubation period of 5 h post-illumination (for flight) to be most optimal. Mock illumination (control) was realized by masking the external wells from light illumination, providing a control population on the same 24-well plate (Figure 1).

Illumination of animals at different wavelengths was realized with a Mai Tai HP with integrated Spectra-Physics 14 W Millennia® pump laser (Newport/SpectraPhysics, Darmstadt, Germany). The tunable wavelengths range from 690 nm to 1040 nm and we used 690, 730, 755, 780, 808, 830, 865 and 900 nm of wavelength. The wavelength range was extended with two laser diodes at 635 nm (Ceralas PDT 635/4W/3 nm/400 um, Ceramoptec GmbH, Bonn, Germany), and 652 nm (Ceralas PDT 652/4W/+−4/400 um, Ceramoptec GmbH, Bonn, Germany).

For rotenone treatment, first instar larvae were grown on 250 µM rotenone until the third instar stage as described [19].

Flight assays were conducted using male flies in batches of 5 flies each. Flies were placed in an empty vial (5×10 cm), gently tapped and scored visually. Flies able to fly were given a score of 1 while those that did not were given a score of 0 [19].

### ATP

ATP levels in adult thoraces or heads was determined as described [13] using an ATP determination kit (Invitrogen, Ghent, Belgium). Luminescence was measured using a luminometer (Biorad, Nazareth Eke, Belgium). The values were normalized to total protein content (Bradford method). To determine ATP production capacity in vitro, we first irradiated flies, waited 5 h and then prepared mitochondrial fractions as described [29]. We incubated 0.25 µg (Bradford method) mitochondrial fraction with malate (1.25 mM), pyruvate (1.25 mM) and ADP (1.25 mM). We first measured ATP levels in the mitochondrial fractions (t0) as described above and waited for 15 or for 30 min and measured ATP levels again in each of the fractions as to assess ATP production capacity within this 15 or 30 min timeframe. The data were normalized to “t0”.

### JC-1

For JC-1 (Molecular probes, Ghent, Belgium) labeling, larval fillets were incubated for 1.5 min with 4 mM JC-1 in HL-3, washed in HL-3 and red and green fluorescence in boutonic mitochondria imaged [7]. Mitochondria in boutons were discerned from others by morphology: the green JC-1 labeling marks bouton outlines. Images of neuromuscular boutons were captured on a Nikon FN-1 microscope with a Clara Andor digital camera, 60x NA 1.0W objective and red over green intensity in the red labeled areas within boutons was quantified using NIS-Elements BR 3.1.

### Immunohistochemistry and Quantification

For immunolabeling, larval fillets were fixed for 20 min in 4% formaldehyde in PBS and permeabilized with 0.4% tritonX100. Brain dissection and whole-mount immunohistochemistry for tyrosine hydroxylase (TH) was performed as described (Wu and Luo, 2006). Primary antibodies used: anti-ATP synthase subunit beta mAb (1∶100; MitoSciences, Cambridge, United Kingdom) and Rabbit anti-TH (1∶100; Chemicon, Darmstadt, Germany). Secondary antibodies: goat anti-mouse alexa 488 and goat anti-rabbit alexa 555 (Invitrogen, Ghent, Belgium). Images were captured with a Zeiss LSM510META confocal microscope and a 63x NA 1.4 oil lens. For larval muscles, images of nuclear regions in muscles 6 and 7 were taken. For TH positive cells in the adult brain, cell bodies in cluster PPM3 were captured (but the phenotypes of mitochondrial morphology were consistent across DA clusters). For quantification of mitochondrial morphology in larval muscles and dopaminergic neurons, the ‘analyzing particle’ plugin of ImageJ was used and rounded mitochondria were automatically detected and their number quantified.

### Oxygen Consumption Measurements

Oxygen consumption was measured with a Clark type oxygen electrode fitted onto an oxygraph (hansatech) apparatus to follow oxygen concentration over time [7]. Mitochondria were isolated from flies [29] and mouse liver [7] as described previously. 10 µg mitochondrial fraction were incubated in EB adapted (200 mM sucrose, 2 mM KH_2_PO_4_ 2 mM MgCl_2_, 10 mM MOPS and 0.1 mM EGTA/Tris) and ADP (300 µM). Complex-specific substrates were added: glutamate (5 mM) and malate (2.5 mM) for Complex I, succinate (5 mM) for Complex II and Ascorbate (6 mM) and TMPD (300 mM) for Complex IV. To inhibit upstream Complexes rotenone (2 mM) was added for Complex II-driven oxygen consumption measurements, antimycin A (0.25 mg/ml) for Complex IV-driven oxygen consumption and potassium cyanide (KCN 3 mM) to block Complex IV. To test if laser light can stimulate oxygen consumption, we applied 808 nm laser light of 25 mW/cm^2^ for 100 s directly onto the mitochondrial suspension and measured the slope of ADP-stimulated oxygen consumption before and after the light stimulus. We calculated the ratio of oxygen consumption after versus before light stimulus. As a control we performed the same treatment but did not switch on the light and calculated the ratio of the slope after versus before ‘mock treatment’.

### Statistical Analysis

All experiments were evaluated using the appropriate ANOVA test followed by post hoc Dunnett test using GraphPad Prism 6.

## Results

### 808 nm Light Stimulation Rescues *pink1* Mutant Flight Defects

Complex IV of the ETC shows absorption maxima around 670 nm and 800–840 nm (see also below) [28], but the higher wavelength penetrates tissues more effectively [30]. We used an 808 nm laser fitted with a frontal light distributor to simultaneously irradiate groups of control (*pink1^RV^*) and *pink1^B9^* null mutant animals (Figure 1A) using different conditions and tested for their ability to fly at different time points following light exposure (Figure 1B–C and Table S1). We find that *pink1^B9^* mutant flies irradiated for 100 s with 25 mW/cm^2^ 808 nm light show rescue of their ability to fly which is most pronounced 5 h following the light stimulation, while no effect was observed in control flies (Figure 1B–C and Table S1). The rescue is only partial but is very similar to the level obtained previously when testing various genetic and pharmacological conditions suppressing *pink1* mutant flight defects [11], [13], [19], [20], [31]. The rescue appears specific to 808 nm light because irradiating *pink1^B9^* mutants using 100 s of 25 mW/cm^2^ 730 nm light that is less absorbed by Complex IV in cells in vitro [32], does not rescue the inability of the mutants to fly and has no effect on control flies (Figure 1D–E). In most of the following experiments we thus used 100 s of 25 mW/cm^2^ 808 nm light to irradiate the flies.

### 808 nm Light Rescues Functional Defects in *pink1* Mutant Mitochondria

**Figure 2:**
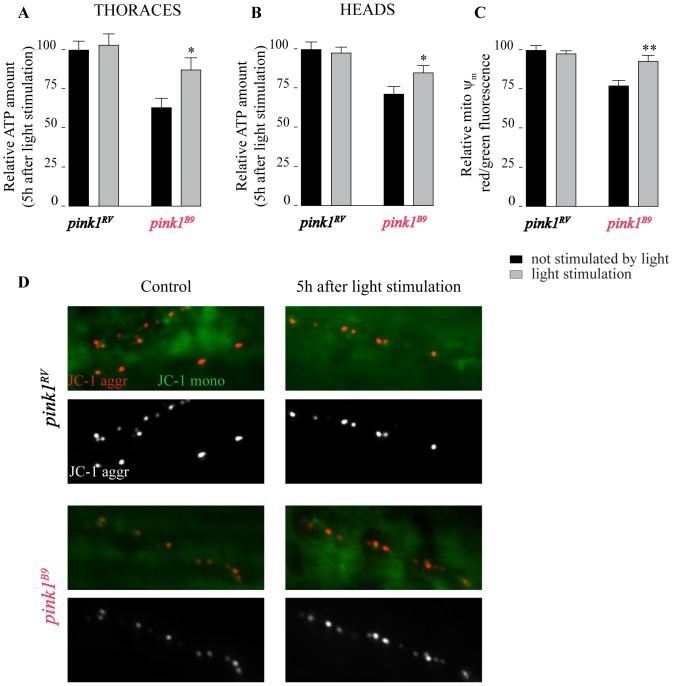
Functional defects in *pink1^B9^* mutant mitochondria are rescued by 808 nm light. A–B- ATP concentration measured in thoracic (A) and head (B) extracts from *pink1^RV^* control and *pink1^B9^* null mutants 5 h after the flies were illuminated (808 nm light stimulation for 100 s at 25 mW/cm^2^; grey bars) or not illuminated (control; black bars) (n = 10 independent assays per condition). C–D- Images (D) and quantification (C) of JC-1 labeling intensity (ratio of red/green fluorescence) as a measure of ψm measured at NMJ synaptic bouton mitochondria of *pink1^RV^* control and *pink1^B9^* null mutant third instar larvae 5 h after flies were illuminated (grey bars) or not illuminated (black bars) (n = 20 synapses per condition). Data in (A–C) are normalized to *pink1^RV^* without illumination ± SEM. ANOVA/Dunnet: *: p<0.05; **: p<0.01.

*Pink1^B9^* mutant flies suffer from mitochondrial dysfunction, including lower ATP levels and reduced mitochondrial membrane potential (ψ_m_) [7], [12], [13]. We irradiated adult mutant and control flies and measured ATP levels 5 h later in neuron-enriched (heads) and muscle-enriched (thoraces) material using a luciferase based assay. Irradiation results in increased ATP levels (Figures 2A–B). Similarly, we assessed ψ_m_ at larval motor neuron endplates 5 h after irradiation using JC-1, a potentiometric dye [33]. JC-1 reports on the ψ_m_ at *Drosophila* motor endplates because in control boutons, red JC-1 fluorescence is clearly visible while in samples treated with the Complex I inhibitor rotenone, red labeling is significantly reduced [19]. Similarly, in samples treated with FCCP, an uncoupler, red labeling is lost [7]. As reported before, red JC-1 fluorescence in *pink1^B9^* larvae is reduced compared to controls (*pink1^RV^*). While JC-1 red/green fluorescence ratio between irradiated and non-irradiated *pink1^RV^* larvae is not appreciably different (Figures 2C–D), red JC-1 fluorescence in irradiated *pink1^B9^* larvae is significantly rescued compared to non-irradiated *pink1^B9^* animals (Figures 2C–D). These data suggest that 808 nm light improves the mitochondrial membrane potential and ATP levels in *pink1^B9^* mutants.

### 808 nm Light Restores Mitochondrial Morphology and this Rescue is Preceded by Functional Rescue

**Figure 3:**
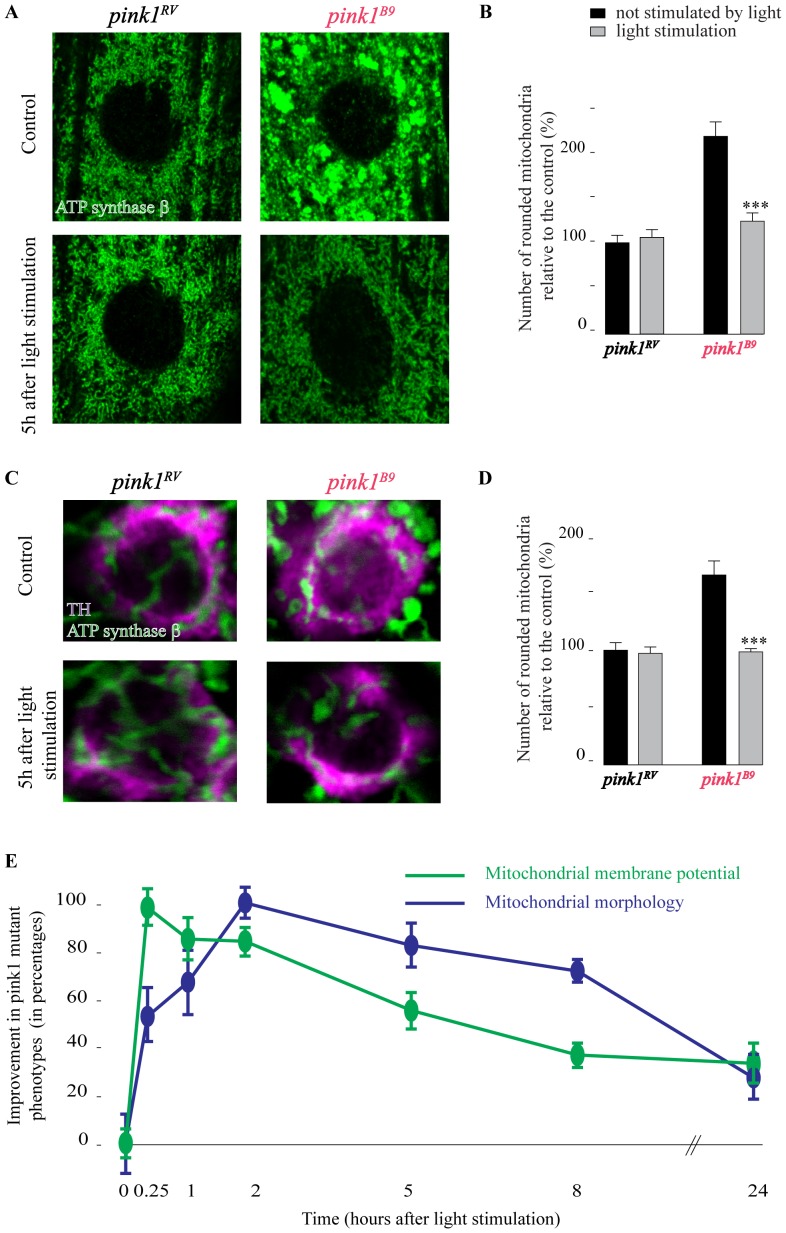
Mitochondrial morphological defects in *pink1^B9^* mutant muscles and DA neurons are rescued by 808 nm light. A–D- Images (A, C) and quantification (B, D) of mitochondrial immunolabeling with Complex V (anti-ATP synthase β) antibody of *pink1^RV^* and *pink1^B9^* mutant muscles (muscles 6/7 in third instar larva) (A, B) and DA neuron cell bodies (PPM3 cluster in adult heads) marked by anti-TH labeling (C, D) 5 h after flies were illuminated (808 nm, 100 s, 25 mW/cm^2^) or not illuminated (control). Quantification of the number of rounded/clumped mitochondria in each condition, normalized to *pink1^RV^* without illumination (B, D). E- Time course of ψ_m_ assessed as the ratio of red/green JC-1 fluorescence labeling intensity measured at mitochondria in boutons of third instar larvae (green) as well as mitochondrial morphology assessed as the density of rounded mitochondria in larval muscle cells (blue) at the indicated time points following illumination and normalized to data obtained at maximum rescue (15 min for JC-1 and 2 h for mitochondrial morphology). n = 20 muscles or synapses (A, B, E) or 10 brains (C, D). Error bars SEM. ANOVA/Dunnet: ***: p<0.001.

A hallmark phenotype in *pink1* null mutant flies is the accumulation of enlarged swollen mitochondria in some cell types including muscle cells (Figures 3A–B) and dopaminergic neuron cell bodies in adult flies (Figures 3C–D) [12], [13]. Light stimulation significantly decreases the number of aggregated and swollen mitochondria (Figures 3A–D), but has no detectable effect on mitochondrial morphology of control animals (Figures 3A–D).

Pink1 might act at different levels causing mitochondrial failure. The protein regulates mitochondrial remodeling and autophagy in a pathway with Parkin [17], [34] and involving remodeling proteins such as Drp1 [31], [35], [36]. On the other hand, Pink1 is also implicated in the control of the ETC [5], [7], [11], [19]. We tested if PBM with 808 nm light impinges on these different mitochondrial pathways. Wild type larvae intoxicated with rotenone, a Complex I inhibitor, show reduced red JC-1 fluorescence [19]. Irradiating these animals with 808 nm light results in a significant improvement of the ψ_m_ measured 5 h post irradiation (Figures S1A–B). Remarkably, mitochondrial defects induced by loss of Parkin or DRP1 are not rescued by 808 nm light (Figures S1C–I). Thus, the data suggest that 808 nm light treatment improves mitochondrial defects induced by pharmacological inhibition of the ETC but not the mitochondrial defects induced by parkin or drp1 loss-of-function mutations. These data may also suggest that the improvement of mitochondrial function and morphology in *pink1* mutants following light stimulation are to some extend caused by effects on ETC performance. We therefore performed a time-course experiment and assessed mitochondrial membrane potential and mitochondrial morphology at different time points following light exposure. Fifteen minutes following irradiation, *pink1^B9^* mutant animals show already a significant rescue of the JC-1 labeling defect that gradually decreases again in subsequent hours (Figure 3E). In contrast, maximum rescue of mitochondrial morphological defects occurs only 2 h after irradiation (Figure 3E). The effect on morphology gradually disappears over a 24 h period. Hence, these data indicate that maximal rescue of *pink1^B9^* defects in mitochondrial function following light stimulation precede, at least in part, the maximal rescue of morphological defects in *pink1^B9^* mutant mitochondria.

### 808 nm Light Facilitates Complex IV-dependent Respiration

**Figure 4:**
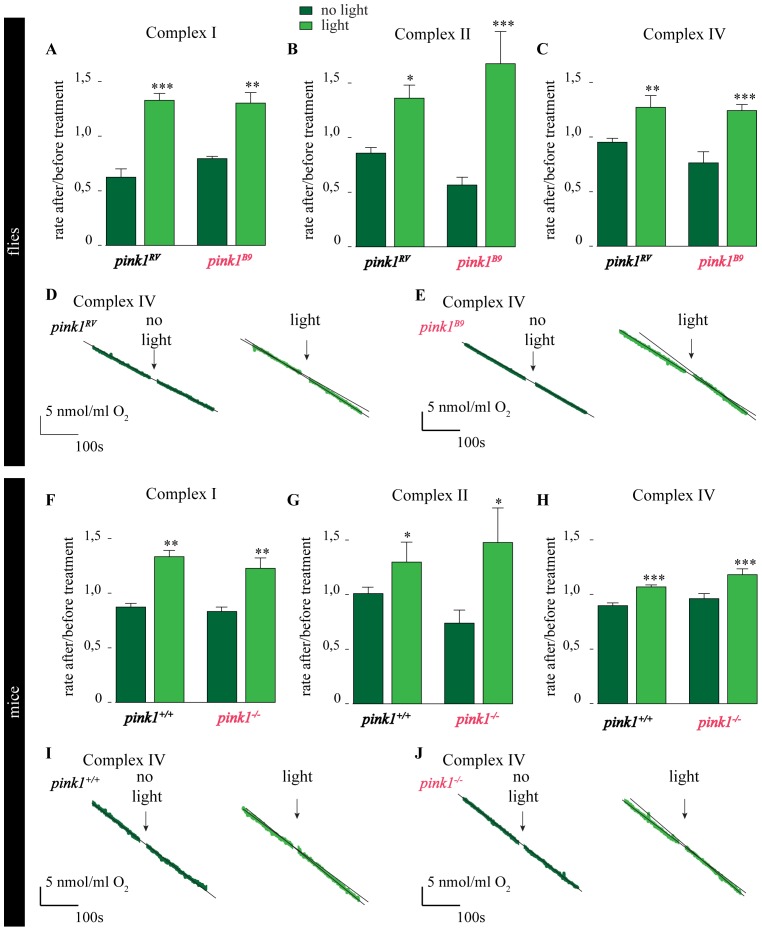
808-dependent respiration. A–E- Quantification of the rate of ADP-stimulated oxygen consumption after versus before light (light green; 808 nm, 100 s, 25 mW/cm^2^) or mock treatment (dark green) when all substrates were present for Complex I- (A) or for Complex II- (B) or for Complex IV- (C) driven respiration in mitochondria isolated from control (*pink1^RV^*) and *pink1^B9^* mutant flies. (D, E) Raw data traces showing O_2_ levels when following Complex IV-driven respiration in control (D) and *pink1^B9^* (E) mutant mitochondria when light (light green) or no light (dark green) were applied. F–J- Quantification of the rate of ADP-stimulated oxygen consumption after versus before light treatment (light green) or mock treatment (dark green) when all substrates were present for Complex I- (F) or for Complex II- (G) or for Complex IV- (H) driven respiration in mitochondria isolated from wild type (*pink1^+/+^*) or *pink1^−/−^* knock out mutant mice mitochondria. Raw data traces showing O_2_ levels when following Complex IV- driven respiration in *pink1^+/+^* (I) and *pink1^−/−^* mitochondria (J) when light (light green) or no light (dark green) were applied. n = 3 independent mitochondrial isolations from flies (A–E) and n = three independent trials from 2 mitochondrial isolations from mouse livers (F–J). Error bars SEM. ANOVA/Dunnet: *: p<0.05; **: p<0.01; ***: p<0.001.

Given that Complex IV of the ETC absorbs 808 nm light in a cellular context [28], we hypothesized that Complex IV may be an important photo-acceptor through which mitochondrial function is improved upon 808 nm PBM. We therefore measured, in real-time, before and after irradiation, oxygen consumption of isolated mitochondria that are energized with Complex-specific substrates, while pharmacologically blocking the immediately upstream complex. Ie, when energizing Complex II, we used rotenone to inhibit Complex I, etc. We first measured Complex I-driven oxygen consumption as basal rate versus ADP stimulated rate. This is reduced in *pink1^B9^* mutants compared to controls, as is consistent with previous data [7]. We then measured the ratio of the ADP-stimulated rate of oxygen consumption after light treatment versus before light treatment. We also performed ‘mock treatments’ where samples were handled identically but the light was not turned on. If light stimulation activates Complex IV, and Complex IV is in part limiting in the ETC, we expect ADP-stimulated oxygen consumption of mitochondria energized with substrates for one of the upstream complexes to be increased as well, since for these measurements electrons are always transferred to reduce oxygen in Complex IV. We find that ADP stimulated Complex IV-driven oxygen consumption following light stimulation is significantly increased in *pink1^B9^* mutant mitochondria and in *pink1^RV^* control mitochondria. Similarly, ADP stimulated oxygen consumption of mitochondria energized with substrates for the upstream ETC complexes is also significantly increased in *pink1^B9^* mutant mitochondria and in *pink1^RV^* control mitochondria following light stimulation (Figures 4A–E). Likewise, mouse mitochondria energized with different ETC substrates also show a significant increase in the oxygen consumption after light stimulation (Figures 4F–J). While light stimulation boosts mitochondrial oxygen consumption in the presence of inhibitors of Complex I or III (rotenone or antimycin; Figures 4B, G and 4C–E, H–J respectively), we do not observe an increase in the oxygen consumption rate following light stimulation in the presence of potassium cyanide, a Complex IV inhibitor (Figure S2A). These data indicate that 808 nm light has an acute effect on ETC Complex IV-driven oxygen consumption. In line with these data, we also find that ATP production of isolated *pink1^B9^* mutant mitochondria is significantly increased if animals are irradiated with 808 nm light (Figure S2B). Hence, 808 nm light boosts Complex IV-dependent mitochondrial oxygen consumption and ATP production capacity.

In a cellular context, Complex IV shows two absorption maxima (around 670 nm and 800–840 nm) in the infra-red light range [28]. To further test if the light-induced rescue of *pink1* mutant phenotypes correlates with this absorption spectrum, we employed a tunable laser set-up to irradiate animals for 100 s with 25 mW/cm^2^ light at wavelengths between 635 nm and 900 nm and assessed JC-1 labeling intensity at NMJ boutons. We find that the reduced red JC-1 labeling in *pink1* mutants is significantly rescued when animals were illuminated with light of wavelengths that are efficiently absorbed by Complex IV, but not when animals were illuminated by light of other wavelengths (Figure S2C), while in control (*pink1^RV^*) flies no difference between different wavelengths is observed. Taken together, these data are consistent with the idea that infra-red light boosts Complex IV dependent respiration and this feature can be used to significantly alleviate the defects in a Pink1 PD model.

## Discussion

In this work we show that PBM using 808 nm light is a strategy that can be followed to alleviate symptoms associated with specific forms of Parkinson’s disease. Our data using genetic models of Parkinson’s disease that affect mitochondrial integrity are also consistent with work showing that PBM protects against the loss of dopaminergic neurons when rodents were intoxicated with the mitochondrial drug MPTP [27]. Combined, our data provide further evidence that mitochondria are central to the mechanism by which PBM yields protective effects. Long wavelength light also appears to harbor positive effects in wound healing, stroke, optic axonal degeneration and ischemic heart injury [23]–[25] and we speculate that also here improved mitochondrial function may contribute to the beneficial properties of PBM that are observed.

Providing one explanation as to how PBM acts on cellular function, we find that near-infrared light stimulates Complex IV-dependent respiration resulting in increased mitochondrial function and improved organismal activity. We show that 808 nm light stimulation was effective in conditions of decreased ETC performance by either toxic (rotenone) or genetic (*pink1*) causes and that it acutely activates Complex IV-dependent oxygen consumption and ψ_m_ in *pink1* mutant mitochondria. Although in control animals Complex IV is acutely activated by 808 nm light, we did not observe an effect on ψ_m_ and ATP levels, suggesting that when sufficient ATP is present, no surplus ATP is being produced. Our data are also suggesting that Complex IV-driven respiration may become limiting in the ETC. In Complex I deficient models, including rotenone treated animals and *pink1* mutants, electrons are still entering the ETC via complex II and inefficiently via Complex I. Facilitating the downstream Complex IV dependent respiration that may be limiting appears to be an efficient strategy to compensate for the genetic or pharmacological defects in Complex I function. That Complex IV may be more limiting than Complex I is also illustrated by the severe defects that are caused by human mutations in Complex IV components in the neonatal period [37], [38].

Our work allows to further elucidate the role of Pink1 in the regulation of mitochondrial function. Pink1 acts with Parkin to mediate mitochondrial remodeling and mitophagy [15], [17] and *pink1* and *parkin* fly mutants show an accumulation of large, swollen mitochondria in some cell types [12], [13]. *Pink1* mutants also show enzymatic defects in Complex I of the ETC [7], [11], [22] but it is unclear if these phenotypes depend on one another. Based on our data, we can now assess the dependency and interplay between these pathways: does light stimulation facilitate mitochondrial remodeling resulting in improved mitochondrial function or vice versa?

Our data argue against the possibility where 808 nm light acts to directly affect mitochondrial remodeling. First, we observe acute increased mitochondrial oxygen consumption upon 808 nm illumination of isolated fly and mammalian mitochondria. Second, mitochondrial fission is a process thought to precede mitophagy; however, 808 nm light does not alter mitochondrial remodeling in illuminated control animals and it does not rescue the accumulation of larger mitochondria seen in *drp1* loss-of-function mutants. Third, 808 nm light was also not effective at alleviating the mitochondrial morphological defects in *parkin* mutants that suffer from defects in mitochondrial remodeling and mitophagy. By contrast, our data supports the notion that 808 nm PBM improves mitochondrial function and suggest that improved mitochondrial function is sufficient to, at least in part, rescue the morphological defects in *pink1* mutant mitochondria. In line, the mitochondrial morphological defects in *pink1* mutants were also rescued upon improving electron transport between NADH and Complex III using expression of the yeast protein NDI1 [11]. Together, these data suggest that mitochondrial dysfunction in *pink1* mutants is an important upstream culprit in the pathological cascade that contributes to different downstream defects, in part including the morphological defects.

Although 800–850 nm light may fully penetrate a fly brain, in human this is not likely to occur. To use infrared light as a therapeutic application in PD patients, implantable devices are being developed that can irradiate selective regions of the affected brain-area(s). Since 808 nm light activates Complex IV both in fly and mammalian mitochondria, our work holds important therapeutic promise with the ultimate goal to improve mitochondrial function in PD patients that suffer from mitochondrial defects.

## Supporting Information

Figure S1 **808 nm light only rescues mitochondrial defects caused by functional defects.** A–B- Images (A) and quantification (B) of JC-1 labeling intensity (ratio of red/green fluorescence) as a measure of ψm, measured at NMJ synaptic bouton mitochondria of wild type (*pink1^RV^*) third instar larvae, placed as first instar larvae on control food or on food supplemented with 250 µM rotenone, 5 h after larvae were illuminated (light stimulation) using 100 s of 808 nm light (25 mW/cm^2^) or not illuminated (control) (n = 20 synapses). C–D- Images of Complex V (anti-ATP synthase β) labeling (C) at control (*y w; FRT40A*) and *drp1^1/2^* mutant muscles (Muscles 6/7 in third instar larval) 5 h after animals were illuminated (808 nm, 100 s, 25 mW/cm^2^) or not illuminated (control). Quantification of the number of rounded/clumped mitochondria in each condition (D) (n = 20 muscles). E–I- ATP concentration measured in thoracic extracts from *park^RV^* control and *park^1/Δ21^* null mutants 5 h after flies were illuminated (light stimulation: 808 nm, 100 s, 25 mW/cm^2^) or not illuminated (control), normalized to *park^RV^* without illumination (E). Quantification (F) and images (G) of JC-1 labeling intensity (ratio of red/green fluorescence) as a measure of ψm measured at NMJ synaptic bouton mitochondria of *park^RV^* control and *park^1/Δ21^* null mutant third instar larvae 5 h after animals were illuminated or not illuminated (n = 20 synapses). Images of Complex V (anti-ATP synthase β) labeling at *park^RV^* and *park^1/Δ21^* mutant muscles (Muscles 6/7 in third instar larval segment A2) 5 h after animals were illuminated or not illuminated (control) (I) and quantification of the number of rounded/clumped mitochondria in each condition (H) (n = 20 muscles). Data are normalized to controls without illumination ± SEM (B, D–F, H). ANOVA/Dunnet: ***: p<0.001; ns = not significant.
(TIF)

Figure S2 **Increased activity of Complex IV leads to increased ATP production.** A- Quantification of the rate of ADP-stimulated oxygen consumption in the presence of the Complex IV inhibitor cyanide after light treatment versus before light stimulation (light green; 808 nm, 100 s, 25 mW/cm^2^) or after mock treatment versus before (dark green) in mitochondria isolated from controls (*pink1^RV^*) and *pink1^B9^* mutant flies. n = 3 independent mitochondrial isolations. Error bars SEM. ANOVA/Dunnet: ns = not significant. B- Time-dependent ATP production in mitochondria isolated from *pink1^B9^* mutant flies that were 5 h earlier illuminated (light stimulation: 808 nm, 100 s, 25 mW/cm^2^) or not (control). ATP produced in vitro was measured after 15 min and after 30 min, normalized to control stimulation after 15 min. n = 10 assays. C- Quantification of JC-1 labeling intensity (ratio of red/green fluorescence) as a measure for Ψ_m_ measured at NMJ synaptic bouton mitochondria of *pink1^B9^* mutant third instar larvae 15 min after flies were illuminated (red bars) with different wavelengths or not illuminated (dark red bar). Dashed line represents mean values of control (*pink1^RV^*) NMJs that were not illuminated; SEM is indicated in gray (n = 20 synapses per wavelength and per condition). Error bars SEM. ANOVA/Dunnett: *: p<0.05; **: p<0.01.
(TIF)

Table S1 **Optimization of light stimulus using the flight capacity of pink1 mutant flies.** Values show the flight percentage ± SEM with different light conditions and tested at different time points after the light stimulation.
(TIF)
