# Supplementary figures and images for: Near-Infrared 808 nm Light Boosts Complex IV-Dependent Respiration and Rescues a Parkinson-Related pink1 Model
Source: PLoS One. 2013 Nov 11;8(11):e78562. doi: 10.1371/journal.pone.0078562 (PMC3823844; doi:10.1371/journal.pone.0078562)

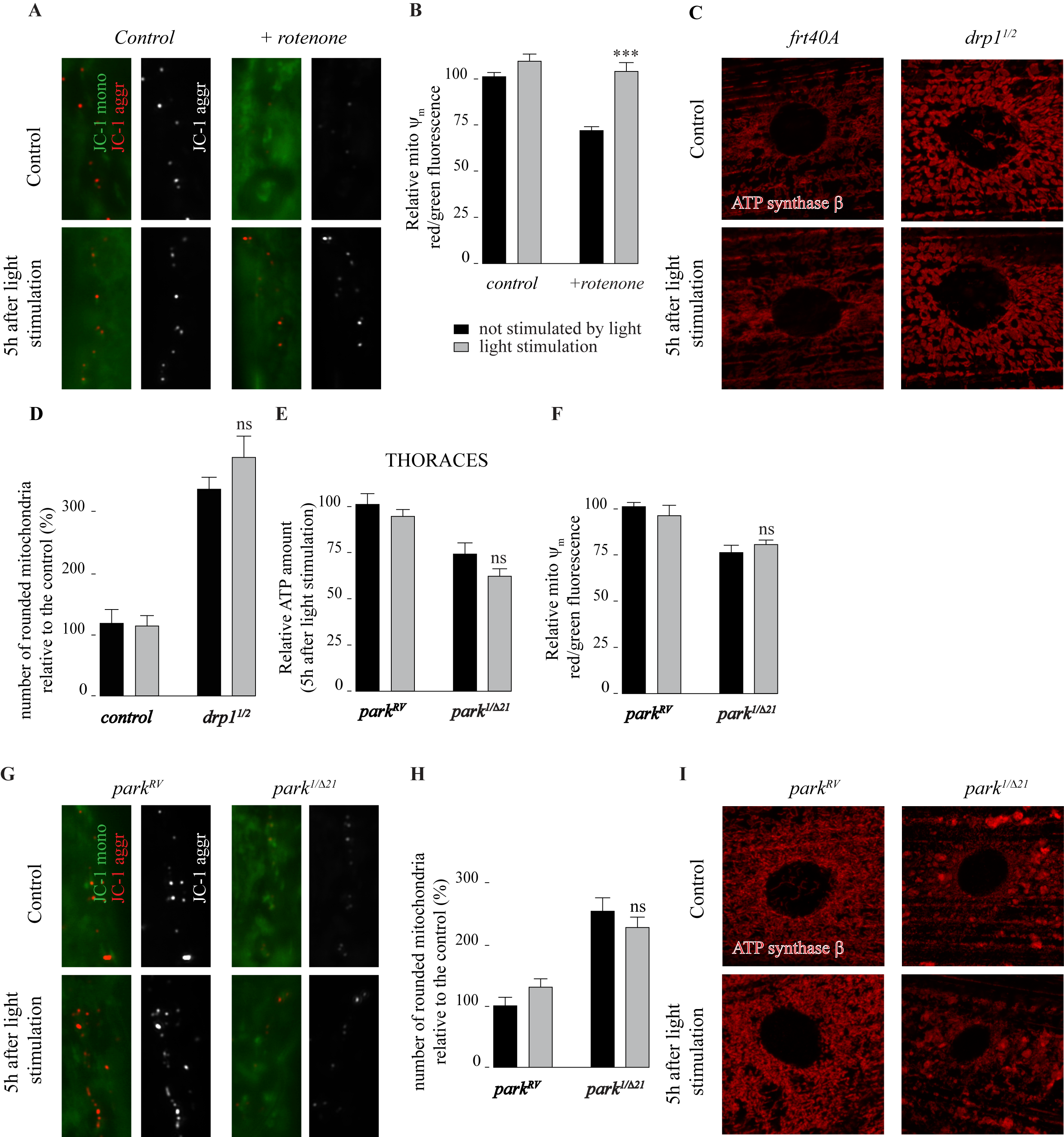

Supplement: Figure S1 — 808 nm light only rescues mitochondrial defects caused by functional defects. A–B- Images (A) and quantification (B) of JC-1 labeling intensity (ratio of red/green fluorescence) as a measure of ψm, measured at NMJ synaptic bouton mitochondria of wild type (pink1RV) third instar larvae, placed as first instar larvae on control food or on food supplemented with 250 µM rotenone, 5 h after larvae were illuminated (light stimulation) using 100 s of 808 nm light (25 mW/cm2) or not illuminated (control) (n = 20 synapses). C–D- Images of Complex V (anti-ATP synthase β) labeling (C) at control (y w; FRT40A) and drp11/2 mutant muscles (Muscles 6/7 in third instar larval) 5 h after animals were illuminated (808 nm, 100 s, 25 mW/cm2) or not illuminated (control). Quantification of the number of rounded/clumped mitochondria in each condition (D) (n = 20 muscles). E–I- ATP concentration measured in thoracic extracts from parkRV control and park1/Δ21 null mutants 5 h after flies were illuminated (light stimulation: 808 nm, 100 s, 25 mW/cm2) or not illuminated (control), normalized to parkRV without illumination (E). Quantification (F) and images (G) of JC-1 labeling intensity (ratio of red/green fluorescence) as a measure of ψm measured at NMJ synaptic bouton mitochondria of parkRV control and park1/Δ21 null mutant third instar larvae 5 h after animals were illuminated or not illuminated (n = 20 synapses). Images of Complex V (anti-ATP synthase β) labeling at parkRV and park1/Δ21 mutant muscles (Muscles 6/7 in third instar larval segment A2) 5 h after animals were illuminated or not illuminated (control) (I) and quantification of the number of rounded/clumped mitochondria in each condition (H) (n = 20 muscles). Data are normalized to controls without illumination ± SEM (B, D–F, H). ANOVA/Dunnet: ***: p<0.001; ns = not significant. (TIF) [file pone.0078562.s001.tif]

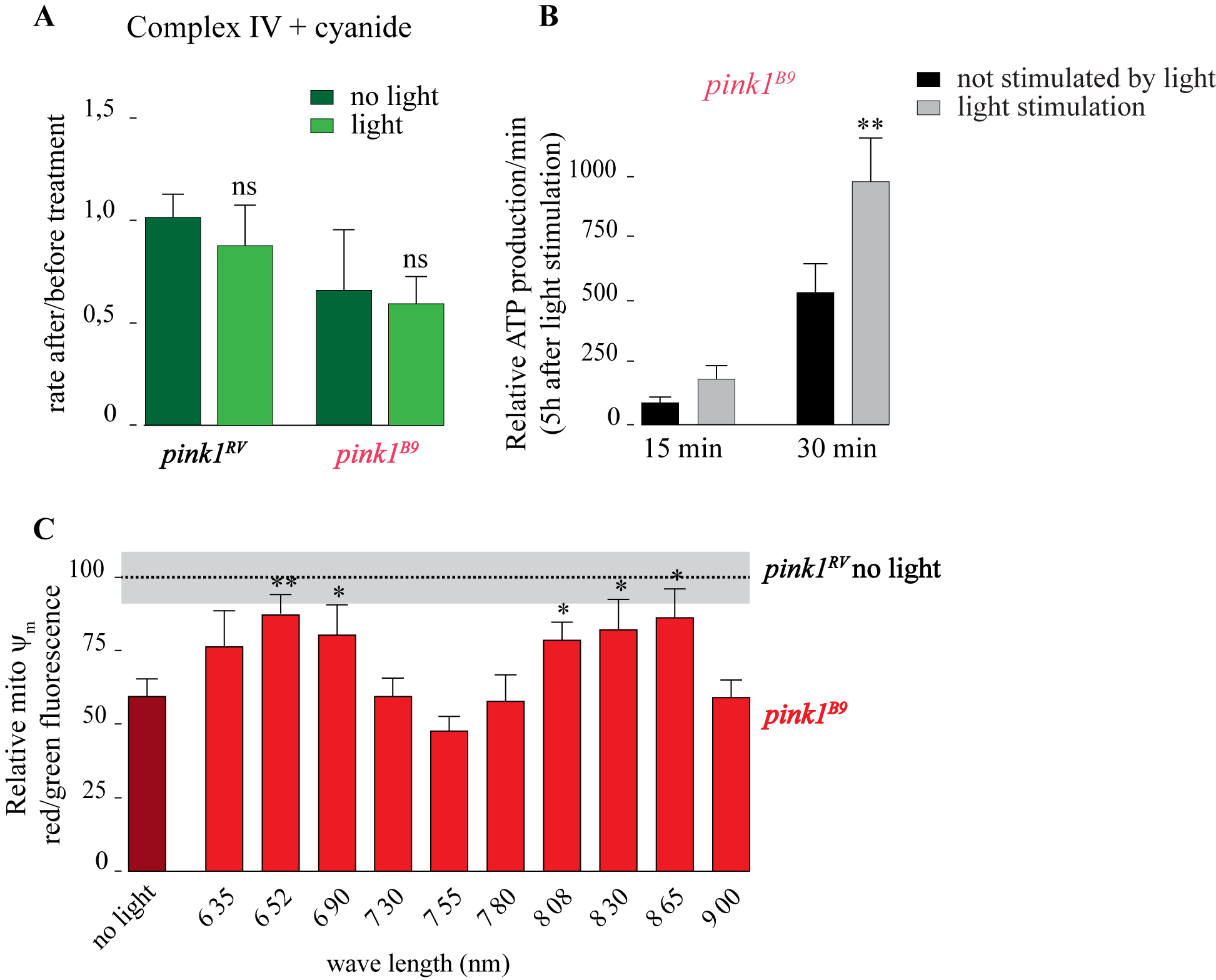

Supplement: Figure S2 — Increased activity of Complex IV leads to increased ATP production. A- Quantification of the rate of ADP-stimulated oxygen consumption in the presence of the Complex IV inhibitor cyanide after light treatment versus before light stimulation (light green; 808 nm, 100 s, 25 mW/cm2) or after mock treatment versus before (dark green) in mitochondria isolated from controls (pink1RV) and pink1B9 mutant flies. n = 3 independent mitochondrial isolations. Error bars SEM. ANOVA/Dunnet: ns = not significant. B- Time-dependent ATP production in mitochondria isolated from pink1B9 mutant flies that were 5 h earlier illuminated (light stimulation: 808 nm, 100 s, 25 mW/cm2) or not (control). ATP produced in vitro was measured after 15 min and after 30 min, normalized to control stimulation after 15 min. n = 10 assays. C- Quantification of JC-1 labeling intensity (ratio of red/green fluorescence) as a measure for Ψm measured at NMJ synaptic bouton mitochondria of pink1B9 mutant third instar larvae 15 min after flies were illuminated (red bars) with different wavelengths or not illuminated (dark red bar). Dashed line represents mean values of control (pink1RV) NMJs that were not illuminated; SEM is indicated in gray (n = 20 synapses per wavelength and per condition). Error bars SEM. ANOVA/Dunnett: *: p<0.05; **: p<0.01. (TIF) [file pone.0078562.s002.tif]

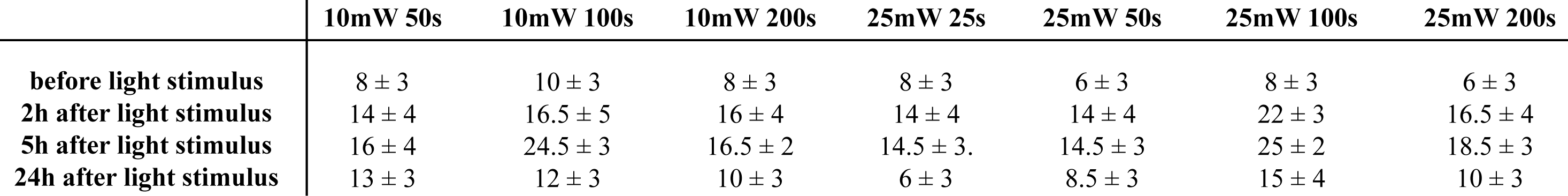

Supplement: Table S1 — Optimization of light stimulus using the flight capacity of pink1 mutant flies. Values show the flight percentage ± SEM with different light conditions and tested at different time points after the light stimulation. (TIF) [file pone.0078562.s003.tif]
